# Post-hospitalization Daycare Treatment for Adolescents With Eating Disorders

**DOI:** 10.3389/fpsyt.2021.648842

**Published:** 2021-05-31

**Authors:** Liron Litmanovich-Cohen, Amit Yaroslavsky, Liron Roni Halevy-Yosef, Tal Shilton, Adi Enoch-Levy, Daniel Stein

**Affiliations:** ^1^Pediatric Psychosomatic Department, Safra Children's Hospital, Sheba Medical Center, Tel Hashomer, Israel; ^2^Hadarim Eating Disorders Outpatient Service, Shalvata Mental Health Center, Hod Hsaharon, Israel; ^3^Sacker Faculty of Medicine, Tel Aviv University, Tel Aviv, Israel

**Keywords:** anorexia nervosa, bulimia nervosa, daycare, day-hospitalization, eating disorders, outcome, remission

## Abstract

**Background:** There are several possible facilities for the treatment of eating disorders (EDs). Specifically, there is the issue of the use of specialized daycare and ambulatory services over inpatient settings and the place of daycare programs following inpatient treatment.

**Aim:** We sought to examine the contribution of post-hospitalization daycare program to the treatment of adolescents hospitalized with an ED.

**Methods:** We assessed 61 female adolescents hospitalized with an ED. All but three were diagnosed with clinical or subthreshold anorexia nervosa (AN). Three were diagnosed with bulimia nervosa. Thirty-seven patients continued with a post-hospitalization daycare program for at least 5 months, whereas 24 did not enter or were enrolled in the program for <5 months. Patients completed on admission to, and discharge from, inpatient treatment self-rating questionnaires assessing ED-related symptoms, body-related attitudes and behaviors, and depression and anxiety. Social functioning was assessed 1 year from discharge using open-ended questions. One-year ED outcome was evaluated according to the patients' body mass index (BMI) and according to composite remission criteria, assessed with a standardized semistructured interview. To be remitted from an ED, patients were required to maintain a stable weight, to have regular menstrual cycles, and not to engage in binging, purging, and restricting behaviors for at least eight consecutive weeks before their assessment.

**Results:** BMI was within normal range at follow-up, whether completing or not completing daycare treatment, and around 75% of the patients had menstrual cycles. By contrast, when using comprehensive composite remission criteria, less than a quarter of former inpatients not entering/not completing daycare program achieved remission vs. almost a half of the completers. In addition, a greater percentage of completers continued with psychotherapy following discharge. Fifty percent of both groups showed good post-discharge social functioning. No between-group differences were found in the BMI and the scores of the self-rating questionnaires at admission to, and discharge from, inpatient treatment.

**Conclusion:** Adolescent females with EDs can maintain a normal-range BMI from discharge to 1-year follow-up, even if not completing daycare treatment. By contrast, completion of a post-hospitalization daycare program may improve the 1-year follow-up ED-related outcome of former ED inpatients.

## Introduction

Eating disorders (EDs), in particular, anorexia nervosa (AN), are psychiatric illnesses with a serious impact, often causing severe distress to patients and families. Less than half of the patients demonstrate full recovery, and the percentages of severe and enduring illness are high (1, 2).

There are currently several possible facilities for the treatment of EDs. Specifically, there is the possibility of using newer forms of specialized daycare and ambulatory services over the more traditional inpatient settings (3). Inpatient treatment is currently suggested for patients with EDs, specifically AN, who are in imminent risk because of their poor physical condition or because of severe suicidal behavior, whose mental state impedes almost completely with their everyday functioning, and whose family, in these specific conditions, is not able to provide the required support (1, 4).

Inpatient care allows for constant supervision and intensive multidisciplinary treatment and, as such, is effective for rapid weight gain (5). Nonetheless, the distinct disadvantages of inpatient care are its high cost and the detaching of the ED patient from his/her family, friends, and school/work system (3, 4, 6, 7).

Indeed, because of these drawbacks, there has been a transition in the past two decades from inpatient to different forms of outpatient programs (1, 8, 9). This process has included the implementation of daycare programs, with a growing preference for daycare vs. inpatient treatment (1, 3, 10–13). This preference stems from both clinical and financial considerations (3, 14). Thus, the costs of daycare programs are less than those of inpatient treatment (3, 12, 15, 16). Two types of daycare treatment exist: halfway in programs, aiming to prevent or reduce the need for inpatient treatment for patients with less severe illness, and halfway out post-hospitalization programs, serving to shorten inpatient treatment and to facilitate the transition from the hospital to the community (5).

In the case of adolescents with EDs, daycare programs assist and maintain independence, support the internalization of skills acquired in therapy, and encourage the use of these skills in daily life (3). During their stay in daycare programs, adolescents can continue with their routines at school, and maintain their social and family roles (5).

Participation in daycare programs requires some degree of cooperation and personal responsibility from the adolescent for his/her own care (15). At the end of the program, the adolescent returns home, is required to cope on his/her own with the complexities of the illness, alongside the support of family, peers, and professionals. Indeed, the period following the discharge from daycare treatment is replete with challenges, including resuming functioning at school and socially, coping with eating, maintaining stable weight, and handling a multitude of emotional problems.

Previous research has mainly focused on the comparison of daycare and inpatient facilities in terms of therapeutic effectiveness and financial viability (10, 15). Only a few studies have specifically addressed the role of daycare programs following inpatient treatment (3, 12, 15, 16). The provision of halfway out daycare programs is highly important for adolescents completing inpatient treatment. First, it maintains a continuity of care and a protective and supportive therapeutic environment (12), allowing for a rapid identification of worsening in the patient's conditions.

Second, post-hospitalization daycare treatment incorporates characteristics of rehabilitation, consistent with psychosocial rehabilitation approaches in mental health care. The goal of psychosocial rehabilitation is to restore the adolescent's ability to live independently within his/her family, create an adequate learning and social environment, and organize effectively the management of his/her treatment. This approach requires a collaboration among patients, families, and treatment providers under the assumption that effective rehabilitation is built on the ability of the youngster to cope with his/her illness and its consequences, and to show at least some motivation for change, and some wish to recover (13, 17–20). In this respect, patients with EDs participating in daycare programs have been found to regard the goal of their treatment not only as reducing the symptoms of their illness, but also as making them capable of sustaining relationships and adopting more functional problem-solving strategies and modes of thinking (21).

Research defines the collaboration of patients with EDs in daycare programs in terms of their cooperation with their nutritional plan (22) and with the overall therapeutic program (23). Nonetheless, these patients may show considerable difficulties in preserving and cooperating with their treatment (19, 24). This is the case for both patients with AN and bulimia nervosa (BN), although the latter may show initial motivation for treatment, to stop their binge/purge behavior (24).

The aim of the present study was to assess the efficacy of a post-hospitalization halfway out daycare program for the treatment of EDs in adolescents. In a previous study of our group (25), assessing 88 female adolescent patients with EDs hospitalized between 2007 and 2012, 51 patients (58%) continued with a daycare program after discharge. Twelve of the 51 patients (23%) treated in this program were rehospitalized from discharge to 1-year follow-up, compared with 16/37 patients (43%) not treated in daycare conditions (difference not statistically significant). These findings urged us to study the effect of post-hospitalization daycare attendance in another sample of female patients treated in our ED inpatient department.

The post-hospitalization halfway out daycare program in our medical center in Israel provides the continuation of nutritional, psychological, psychosocial, and psychiatric care to adolescents with EDs completing inpatient treatment. Participation in this program is voluntary, recommended by the inpatient treatment team to all patients and families who are willing to enroll in this treatment and who live close enough to our center (i.e., there are no specific inclusion and exclusion criteria).

The present study examined the contribution of post-hospitalization daycare treatment to remission from an ED at 1-year post discharge from inpatient treatment in adolescents continuing with the program for at least 5 months following their discharge from inpatient treatment. This group was compared with patients discharged from inpatient treatment who did not enter the program or continued it for <5 months. This cutoff point was chosen by the working team of the daycare program because of their clinical impression that the adherence of the adolescents to the different group treatments offered in daycare increased after that time, while the risk of leaving the program prematurely decreased. In addition, it was relatively similar to the mean duration of attendance to the daycare program in our previous study (6.2 ± 2.5 months; 25).

The following were our hypotheses:

1. More patients staying in daycare program for at least 5 months will be remitted from their ED according to the remission criteria of the present study, in comparison with patients not entering the program or completing <5 months of treatment.
2. In addition, patients completing the daycare program, in comparison with patients not entering the program or not completing it, would show at 1-year post-discharge follow-up higher body mass index (BMI), higher rate of menstruation, and better social functioning.
3. Patients choosing to cooperate with and complete our daycare program would be different from non-completers in showing less severe eating pathology and body image disturbances, less severe depression and anxiety, and higher BMI, at both admission to and discharge from inpatient treatment (the point of entrance to daycare), as well as shorter duration of inpatient treatment [all the parameters described in hypotheses (2) and (3) were previously shown to be associated with remission from an ED (25–30)].

## Methods

### Population

The design of this study was prospective and longitudinal. The research population included 61 female adolescents hospitalized between 2013 and 2017 because of an ED, at the Pediatric Psychosomatic Department, Safra Children's Hospital, Sheba Medical Center, Tel Hashomer, Israel. They represented a different group of patients from that described in our previous study about factors associated with remission from EDs (25). All girls were offered by the department's treatment team to continue with the daycare program following their discharge from inpatient treatment. Thirty-seven girls continued with the daycare program for at least 5 months. Fourteen girls did not enter the program [either living in a too distant place in Israel to be able to visit the program regularly (*n* = 8) or choosing not to enter the program (*n* = 6)], and 10 girls stayed in the program for <5 months. No differences were found between the girls not attending and not completing the program in any of the study measures introduced. Thus, owing to the small number of participants in each subgroup, we combined them to one group of 24 non-completers. The exact description of the patients included in the study is found in our flowchart.

Criteria for inclusion in the study were (1) female gender, (2) over the age of 15 years, (3) the index hospitalization was the first in our setting, (4) a good understanding of the Hebrew language, (5) parents and patients agreeing to participate in the study, including in the follow-up assessment, (6) completing inpatient treatment, and (7) living near enough to the hospital to be able to continue with the day care program if they so wished. Exclusion criteria were lifetime or current schizophrenic spectrum disorders, bipolar disorder (it is the policy of this department not to hospitalize patients with these comorbid disturbances), organic brain disorder, intellectual disability, and lifetime or current medical illnesses that could potentially affect appetite or weight (e.g., diabetes mellitus or thyroid disorders).

Participants and parents, in the case of minors under the age of 18, signed a written informed consent, after being explained about the aims of the study. The study was approved by the Ethics Review Board of the Sheba Medical Center, Tel Hashomer.

### Description of Inpatient and Post-Hospitalization Halfway Out Daycare Treatment in Our Facility

The pediatric ED treatment service at the Safra Children's Hospital, Sheba Medical Center, Tel Hashomer, Israel, includes inpatient, daycare, and ambulatory programs for children and adolescents between the ages 6 and 18 years with diverse ED types. Treatment is provided by a multidisciplinary team at varying levels of intensity, depending on the severity of the ED and comorbid disorders, and the overall functioning of the patient's family. The center is a “wrap-around” service, i.e., patients can move from one facility to the other, according to their condition.

During inpatient treatment, patients receive multimodal treatment interventions tailored for the treatment of the ED, comorbid psychiatric disorders, and different psychosocial difficulties. Upon discharge, patients are offered a daycare program in the afternoon hours, to allow for their reintegration into the school system. When considered stabilized, patients are referred to our ambulatory service.

The integrative treatment protocol in our service corresponds with other structured programs for adolescents with AN or BN (31, 32). The protocol includes the following: a behaviorally oriented nutritional rehabilitation program with structured meal supervision, either individual psychodynamically oriented psychotherapy or individual cognitive behavioral therapy (CBT; depending on the specific illness and the aims of treatment), individual expressive movement therapy, family therapy [either family-based therapy (33) or systemic family therapies (34)] or parental consultation, psychodynamic group therapy, CBT group sessions [“classical” CBT (35) and cognitive–motivational treatment based on the Maudsley Model of Anorexia Nervosa Treatment for Adults (MANTRA) protocol (36)], group expressive movement therapy, and parents' group. The inclusion of psychodynamic psychotherapy in the treatment regimen is designed to address intrapsychic and interpersonal developmental needs of adolescents often burdened with long-standing illness, in addition to the specific ED-related therapies administered (37). It is of note that other daycare programs in patients with AN have used psychodynamic psychotherapy as their main treatment, showing favorable results (38). While inpatient treatment includes all these therapeutic modalities, daycare treatment includes only some of these interventions, as required, but nutritional consultation, individual psychotherapy family therapy/parental consultation, and different types of group therapies are usually maintained.

The daycare facility, located in the Safra Children's Hospital near the inpatient department, operates three times a week, during the afternoon hours, to enable school attendance. Patients eat two supervised meals, one in the daycare dining room and one in the hospital's cafeteria, to be familiarized with the normal eating of other customers. The staff of both inpatient and daycare programs includes child and adolescent psychiatrists, registered nurses, clinical nutritionists, clinical psychologists, movement therapists, psychology students supervising the meals, and a school program (only for inpatients).

### Assessment

The diagnosis of AN, BN, and eating disorders not otherwise specified (EDNOS) and the diagnosis of comorbid psychiatric disorders (including depressive disorders, anxiety disorders, and obsessive–compulsive disorders) have been established according to the DSM-IV criteria (39), using the Structured Clinical Interview for DSM-IV Axis I Disorders—Patient Edition [SCID-I/P Version 2.0; (40)]. We have decided not to diagnose our patients according to the DSM 5 criteria (41) because the remission criteria used in our study are based on the DSM-IV (39). Highly experienced child and adolescent psychiatrists (DS, AY, and AEL) have assessed independently all patients. All diagnoses have been confirmed in clinical meetings of the department's team. Only patients for whom there has been a unanimous agreement of their ED diagnosis could enter the study. Baseline demographic data, admission and discharge BMI, and admission menstruation data, have been obtained from the patients' medical files.

### Dependent Variables

#### Physiological Measures

BMI, defined as body weight in kilograms divided by height in meters squared, was assessed at admission to, and discharge from, inpatient treatment, as well as at 1-year follow-up from discharge. Amenorrhea, defined according to the criteria of the DSM-IV (39) as the absence of at least three consecutive menstrual periods following menarche, was assessed at 1-year follow-up according to self-report. All patients had evidence of amenorrhea either at admission or at some time before their admission to inpatient treatment.

#### Self-Rating Scales

1. Maladaptive eating-related parameters were assessed using the 26-item Eating Attitude Test-6 [EAT-26; (42)], previously shown to differentiate Israeli ED patients from non-ED controls (25). Scores ≥20 indicated the existence of disordered eating, whereas scores <20 were considered to indicate lack of disordered eating (42). The internal consistency of the EAT-26 in the present study was α = 0.90.
2. Depression was assessed using the 21-item Beck Depression Inventory [BDI; (44)]. The BDI has been previously used in ED patients (43), including in Israeli samples (25, 29). Scores ≤ 19 indicated the absence of depression, whereas scores >20 indicated the presence of depression (44). The internal consistency of the BDI in this study was α = 0.87.
3. Anxiety was assessed using the 40-item State–Trait Anxiety Inventory [STAI; (45)], measuring the severity of anxiety at the time of examination (STAI—State), and the general tendency to display anxiety (STAI—Trait). The STAI was previously used in ED patients (43), including in Israeli samples (25, 29). Scores ≤ 40 indicated the absence of trait and state anxiety, whereas scores >41 indicated the presence trait and state anxiety (45). The internal consistency of the STAI—State and STAI—Trait scales in this study was α = 0.92 and α = 0.93, respectively.
4. The Body Investment Scale [BIS; (46)] is a 24-item self-rating scale used to measure the degree of emotional investment in the body and body experience in four aspects (each containing six items): feelings and attitudes about the body (e.g., “*I hate my body*.”), comfort in physical touch (e.g., “*I feel uncomfortable when people get too close to me physically*.”), body care (e.g., “*I believe that caring for my body will improve my well-being*.”), and body protection (e.g., “*It makes me feel good to do something dangerous*.”). The final score of the BIS is calculated by summation of the four separate scales. A higher score indicates more positive feelings toward the body, greater comfort with touch, and greater body protection and body care. The BIS has been previously used in patients with EDs (47). Scores ≤ 12 indicate disturbances in body investment, whereas scores >13 indicate healthy body investment (46). The internal consistency of the different BIS scales in the present study has been α = 0.90, α = 0.86, α = 0.89, and α = 0.91, for feelings and attitudes about the body, comfort in physical touch, body care, and body protection, respectively. In the present study, we have used only the total BIS score, comprised of the sum of the scores of all individual BIS scales (46).

#### ED-Related Remission Criteria

We applied the remission criteria proposed by Strober (26) and Herzog (48) for AN, and by Herzog (48) and Keel (49) for BN. This replicated the criteria applied in our previous study of remission from EDs (25). Accordingly, to be remitted from AN-restricting type (AN-R), or EDNOS-restricting type (EDNOS-R), patients were required to maintain a stable weight of over 85% of ideal body weight (IBW), to have regular menstrual cycles, and not to engage in binging, purging, or restricting eating patterns for at least eight consecutive weeks prior to the assessment. For the assessment of IBW, we used the data of the Centers for Disease Control and Prevention (2000) Growth Charts (www.cdc.gov/growthcharts) found adequate also for Israeli children and adolescents (50). To be remitted from BN, patients were required to be abstinent from binging, purging, and restricting eating patterns for at least eight consecutive weeks prior to the assessment. To be remitted from AN-binge/purge (B/P) type, or from EDNOS-B/P, patients were required to fulfill both criteria for at least eight consecutive weeks prior to the assessment.

In line with previous recommendations (26), we have further divided the criteria for ED-related remission into complete and good remission. Accordingly:

1. Complete remission: All required behavioral remission criteria and participants do not demonstrate maladaptive eating-related preoccupations. This is defined as ED-related preoccupations occurring for not more than 30 min daily.
2. Good remission: All required behavioral remission criteria, but participants demonstrate maladaptive ED-related preoccupations. This is defined as ED-related preoccupations occurring for more than 30 min daily.
3. The non-remitted patients have been divided into:
4. Intermediate outcome: For patients with AN or EDNOS-R: Weight is less than 85% of IBW, or menstrual cycles are irregular or absent, or there is evidence of maladaptive eating behaviors [i.e., not meeting DSM-IV (39) criteria for full-blown AN].
5. For patients with BN and EDNOS-B/P: evidence of subsyndromal B/P behaviors [i.e., not meeting DSM-IV (39) criteria for full-blown BN].
6. Poor outcome: Participants meeting the DSM-IV criteria for full-blown AN, BN, or EDNOS.

For the purposes of this study, because of the relatively small number of participants, we combined patients belonging to criteria (1) or (2) to represent remission from an ED and patients belonging to criteria (3) or (4) to represent nonremission from an ED.

#### Social Functioning

In line with our previous study (25), social functioning at 1-year follow-up was evaluated with open questions. Poor social functioning was defined as having poor relations with family and/or peers, spending time mostly alone, with no motivation to renew old or create new friendships. Intermediate functioning was defined as some contact with family and/or peers, and some motivation to renew old or create new friendships. Good social functioning was defined as having good relations with family and/or peers, meeting friends occasionally, and having good motivation to renew old or create new friendships. Finally, very good social functioning was defined as having meaningful and fulfilling relationships with family and peers, old and/or new, spending a considerable amount of time with others, and/or being involved in a romantic relationship.

Social functioning was rated on a four-point scale, where (1) represented poor and (4) represented very good functioning. In keeping with the time duration required for the definition of remission from an ED, very good or good functioning was defined if present for at least eight consecutive weeks prior to the follow-up assessment. Otherwise, it was defined as intermediate or bad.

### Procedure

Patients (and parents in the case of minors) were contacted around 1-year post-discharge. Those agreeing to participate in the follow-up assessment were included in the study. Remission from an ED according to Strober's criteria was assessed using the Eating Disorders Family History Interview (EDFHI) (51). This is a semistructured clinical interview designed to gather detailed information about weight and eating history previously used in studies of ED patients (52), including in Israeli samples (25, 29). The EDFHI allows for a detailed assessment of current, minimal, and maximal body mass index (BMI), menstrual history, lifetime, and current restricting, binging, and purging behaviors, and the extent of preoccupation with eating, weight, and body image-related issues and of maladaptive physical exercising.

Master's level psychology and social work students administered the EDFHI. All were blind to the ED diagnosis of the patients at admission, and whether the patients attended, or did not attend, the daycare program. These students were trained in psychiatric interviewing by the study's principal investigator (DS). The degrees of inter-rater reliability between these interviewers and the principal investigator for the EDFHI (according to the correlation coefficient procedure) was *r* = 0.89–0.91.

These students also distributed the self-rating questionnaires at admission, discharge, and 1-year follow-up, and assessed the patients' psychosocial functioning at follow-up with open-ended questions. Thereafter, they assessed with open-ended yes/no questions whether the patients entered the daycare program following their discharge from inpatient treatment. If the response was positive, they assessed the duration (in months) of daycare treatment. Patients were also asked with open-ended yes/no questions whether they continued with psychotherapy/pharmacotherapy following their discharge from inpatient treatment.

Weight and height were taken last in all participants by the registered nurse of the daycare program during the morning hours according to accepted criteria (53). Weight and height were similarly measured on admission to, and discharge from, inpatient treatment.

### Statistical Analysis

Adolescents staying in the program for at least 5 months were compared with the adolescents who ether did not enter the program or did not complete at least 5 months of daycare treatment. The analysis of between-group differences in categorical variables at 1-year follow-up (Strober's remission criteria, regularity of menstruation, psychosocial functioning, and consistency of treatment) as well as of the type of ED at admission was performed using a non-parametric chi-square test of independence. The analysis of between-group differences in continuous variables (BMI, EAT-26, BDI, STAI—State and trait, and BIS) at admission, discharge, and 1-year follow-up was performed using analysis of variance (ANOVA) with repeated measures. Between-group differences in age at admission, duration of illness before admission, and duration of inpatient treatment were assessed using *t-*tests for independent measures. We used the Statistical Package for Social Sciences software, version 21.0 for Windows.

## Results

### Background Data

Table 1 summarizes the between-group differences in the background data. No differences were found for age and duration of illness and of inpatient treatment. Table 2 summarizes the between-group differences in diagnosis and treatment at admission. No differences were found for type of ED, comorbid psychiatric diagnoses, and psychopharmacotherapy. Specifically, more than half of the patients were diagnosed with AN at admission to inpatient treatment. Moreover, all patients with EDNOS were diagnosed with subthreshold AN (39). Only three patients were diagnosed with BN. Around two thirds of the patients had evidence of a comorbid psychiatric disorder at admission, and around a half were treated at that time with psychotropic medications [mostly serotonin-specific reuptake inhibitors (SSRIs)].

**Table 1:** Between-group differences in background data.

	**Adolescents staying in the daycare program for at least 5 months (*n*=37)**		**Adolescents not entering the daycare program or not completing at least 5 months of treatment (*n* = 24)**		***t*(1, 60), *p***
**Variable**	**M**	**SD**	**M**	**SD**	
Age (years)	16.35	1.35	16.53	1.50	*t* = −0.486, *p* = 0.629
Duration of illness prior to admission to inpatient treatment (years)	1.90	1.48	2.56	2.26	*t* = −1.369, *p* = 0.176
Duration of inpatient treatment (months)	10.31	15.71	6.55	3.19	*t* = 1.108, *p* = 0.273

**Table 2:** Between-group differences in diagnosis and treatment at admission to inpatient treatment.

	**Adolescents staying in the daycare program for at least 5 months (*n* = 37)**	**Adolescents not entering the daycare program or not completing at least 5 months of treatment (*n* = 24)**	**χ^2^, *p***
Variable			
ED Diagnosis			χ^2^(2) = 0.600, *p* = 0.741
Anorexia nervosa	24 (64.70)%	14 (56.52%)	
Bulimia nervosa	2 (5.88%)	1 (4.35%)	
Eating disorders not otherwise specified (EDNOS)^*^	11 (29.42%)	9 (39.13(%	
Psychiatric comorbidity^**^			χ^2^ (1) = 0.44, *p* = 0.833
Yes	24 (63.64%)	15 (60.87%)	
No	13 (36.36%)	9 (39.13%)	
Psychopharmacological treatment prior to admission to inpatient treatment			χ^2^ (1) = 0.261, *p* = 0.609
Yes	20 (54.84%)	11 (47.62%)	
No	17 (45.16%)	13 (52.38%)	

*ED, eating disorder;*

* *all patients with EDNOS were diagnosed with subthreshold anorexia nervosa;*

** *comorbidity included depressive disorders, anxiety disorders, and obsessive-compulsive disorder*.

### Between-Group Differences at 1-Year Follow-Up

Table 3 summarizes the between-group differences for remission, social functioning, and consistency in treatment at 1-year post-discharge follow-up. Almost half of the patients completing treatment in our post-hospitalization daycare center were considered remitted at 1-year follow-up according to Strober's criteria, compared with less than a quarter of patients not receiving full daycare treatment, this difference being significant. Social functioning was rated as good or very good [categories (3) and (4)] in about half of the patients of both groups, and bad or very bad [categories (1) and (2)], in the other half with no between-group differences.

**Table 3:** Between-group differences in ED outcome, the consistency of treatment and social functioning at one-year follow-up.

	**Adolescents staying in the daycare program for at least 5 months (*n* = 37)**	**Adolescents not entering the daycare program or not completing at least 5 months of treatment (*n* = 24)**	**χ^2^(1)**	***p***
Strober's remission criteria			3.98	*p* = 0.046
Not remitted from an ED	20 (54.1%)	19 (79.2%)		
Remitted from an ED	17 (45.9%)	5 (20.8%)		
Consistency in psychotherapeutic treatment^a^			6.01	*P* = 0.014
Yes	34 (91.67%)	16 (66.67%)		
No	3 (8.33%)	8 (33.33%)		
Consistency in psychopharmacological treatment^b^			0.14	*p* = 0.907
Yes	27 (72.22%)	17 (70.83%)		
No	10 (27.78%)	7 (29.17%)		
Social functioning				
Good functioning	18 (50%)	18 (50%)	0	*p* = 1.00
Bad functioning	12 (50%)	12 (50%)		

a *All patients were recommended psychotherapy at discharge from inpatient treatment.*

b *All patents were recommended psychopharmacotherapy at discharge from inpatient treatment.*

ED, eating disorder.

All patients were treated with psychotropic medications (mostly SSRIs) at their discharge from inpatient treatment, and all were suggested at that time to continue with psychotherapy and pharmacotherapy. More than two thirds of the patients in both groups continued with pharmacotherapy at follow-up (mostly SSRIs), with no between-group differences (see Table 3). By contrast, we found a significant between-group difference with respect to psychotherapy. Specifically, almost all adolescents staying in the daycare program for more than 5 months continued with psychotherapy in comparison with around two thirds of the non-completers (see Table 3). Most of the patients in both groups were treated at follow-up with psychodynamic psychotherapy.

Table 4 summarizes the between-group differences in BMI at the three assessment points and in the self-rating scales at admission to, and discharge from, inpatient treatment. Whereas, all patients have responded to these questionnaires at admission and discharge, only about a half have completed them at follow-up, thus, not enabling the inclusion of the follow-up data in the multivariate analysis. Regarding the findings for the BMI, it is of note that while two of the 37 patients in the completers group and one of the 24 patients in the non-completers group have been diagnosed with BN (see Table 3), all other patients have been diagnosed with clinical or subthreshold AN according to the DSM-IV (39). This suggests that the findings for the BMI are likely meaningful.

**Table 4:** Between-group differences in BMI, eating-related symptomatology, depression, anxiety, and body image at the different study time points.

	**Adolescents staying in the day-hospital program for at least 5 months (*n* = 37)**	**Adolescents who did not enter the daycare program or did not stay in it for at least 5 months (*n* = 24)**	Time*day-treatment interaction effect	**Day-treatment main effect**	**Time main effect**
BMI			*F*_(2, 59)_ = 1.432, *p* = 0.237	*F*_(2, 59)_ = 0.045, *p* = 0.833	*F*_(2, 59)_ = 58.051 *p* < 0.001
Admission to inpatient treatment	M = 16.56, SD = 2.02	M = 16.97, SD = 3.05			
Discharge from inpatient treatment	M = 20.12, SD = 0.81	M = 20.45, SD = 1.67			
One-year follow-up	M = 19.82, SD = 1.61	M = 19.35, SD = 1.72			
Eating attitudes test (EAT-26)			*F*_(1, 60)_ = 0.608, *p* = 0.440	*F*_(1, 60)_ = 0.158, *p* = 0.693	*F*_(1, 60)_ = 8.219 *p* = 0.006
Admission to inpatient treatment	M = 43.19, SD = 18.64	M = 38.83, SD = 15.87			
Discharge from inpatient treatment	M = 31.74, SD = 16.51	M = 32.28, SD = 27.06			
Depression (BDI)			*F*_(1, 60)_ = 0.094, *p* = 0.760	*F*_(1, 60)_ = 1.024, *p* = 0.317	*F*_(1, 60)_ = 9.970, *p* < 0.003
Admission to inpatient treatment	M = 30.68, SD = 14.64	M = 27.24, SD = 13.03			
Discharge from inpatient treatment	M = 24.97, SD = 14.18	M = 20.29, SD = 17.87			
State anxiety (STAI-State)			*F*_(1, 60)_ = 1.637, *p* = 0.207	*F*_(1, 60)_ =.176, *p* = 0.677	*F*_(1, 60)_ = 5.337 *p* = 0.025
Admission to inpatient treatment	M = 57.29, SD = 10.82	M = 54.74, SD = 11.32			
Discharge from inpatient treatment	M = 53.79, SD = 9.42	M = 49.68, SD = 14.15			
Trait anxiety (STAI-Trait)			*F*_(1, 60)_ = 1.003, *p* = 0.321	*F*_(1, 60)_ =.394, *p* = 0.533	*F*_(1, 60)_ = 6.223, *p* = 0.016
Admission to inpatient treatment	M = 56.24, SD = 11.18	M = 56.58, SD = 9.83			
Discharge from inpatient treatment	M = 53.47, SD = 9.74	M = 50.11, SD = 11.70			
Body investment scale (BIS)			*F*_(1, 60)_ = 1.155, *p* = 0.288	*F*_(1, 60)_ = 0.653, *p* = 0.423	*F*_(1, 60)_ = 0.000 *p* = 0.991
Admission to inpatient treatment	M = 11.90, SD = 2.51	M = 11.99, SD = 1.70			
Discharge from inpatient treatment	M = 11.56, SD = 1.81	M = 12.33, SD = 2.44			

*BMI, body mass index; EAT-26, Eating Attitudes Test-26; BDI, Beck Depression Inventory, STAI, State Trait anxiety Inventory; BIS, Body Investment Scale*.

According to Table 4, the BMI of the patients in both groups improved significantly between admission to, and discharge from, inpatient treatment, being maintained at 1-year post-discharge follow-up. Both groups showed at that time mean BMIs within normal range, i.e., BMI = 19.82 ± 1.61 kg/m^2^ in completers vs. BMI = 19.35 ± 1.72 kg/m^2^ in non-completers (see Table 4). No between-group difference was found for BMI. Similarly, no between-group differences were found in the presence of menstruation at follow-up [χ^2^(1) = 0.65, *p* = 0.41]. Specifically, 32 patients (86%) completing the daycare program reported regular menstruation in comparison with 18 patients (75%) not attending/not completing the program.

No between-group differences were found in ED-symptomatology, attitudes and behaviors toward the body, and depression and anxiety both at admission to, and discharge from, inpatient treatment. Nonetheless, an improvement in the scores of all scales except for BIS was found from admission to discharge (see Table 4). Despite this improvement, the means of the scores showed that the patients in both groups still showed at discharge symptoms compatible with disturbed eating on the EAT-26 (mean EAT-26 score of both groups >20), depression on the BDI (mean BDI score of both groups >19), elevated anxiety (state and trait) on the STAI, (means STAI scales scores for both groups >40), and disturbed attitudes and behaviors toward the body on the BIS (mean BIS score for both groups <11; Table 4). It is unfortunate that we did not have the findings for these scales at follow-up to see whether a normalization in these measures would be found at that time and whether the improvement would be greater in the patients completing daycare.

## Discussion

The aim of the present study was to examine the contribution of daycare treatment, as an add-on follow-up program to inpatient care, to the post-discharge 1-year outcome of female adolescents hospitalized because of an ED. In keeping with our first hypothesis, more adolescents staying in the daycare program for at least 5 months, in comparison with those not entering the program or completing <5 months of treatment, were defined as remitted from their ED according to Strober's (26) criteria. Thus, almost half of the patients defined as completers vs. less than a quarter of the non-completers were able to maintain a stable weight of over 85% of IBW, to have regular menstrual cycles, and not to engage in binging, purging, or restricting eating patterns for at least eight consecutive weeks before the 1-year post-discharge assessment (see Table 3). By contrast, the second hypothesis was not confirmed, in that no between-group differences were found at follow-up for BMI, presence of menstruation, and psychosocial functioning. Only adherence to psychotherapy was found to differentiate between the two groups, with more than 90% of the patients completing daycare treatment continuing with psychotherapy in comparison with two thirds of the non-completers (see Table 3). The third hypothesis was also not confirmed, in that no differences were found between patients completing and not entering/not completing our daycare program in BMI and severity of ED-related symptoms, body-related attitudes, and depression and anxiety, both at admission to, and discharge from, inpatient treatment.

Several aspects should be considered in the analysis of our findings. First, although relatively young, the female adolescents with EDs in both groups presented a relatively severely ill population with more than 2 years of illness prior to hospitalization, a high rate of psychiatric comorbidity at admission, likely requiring psychopharmacological intervention, and mean inpatient treatment of more than 6 months (see Tables 1, 2). Second, there were no between-group differences in any of the prehospitalization or inpatient parameters assessed [i.e., age, duration of illness before admission, BMI, duration of inpatient treatment (see Table 1), type of ED, ED-related symptomatology, psychiatric comorbidity (according to both DSM-IV diagnoses and self-rating questionnaires), and psychopharmacological treatment (see Tables 2, 4)]. Similarly, there were no between-group differences in BMI and the scores of the self-rating questionnaires at discharge from inpatient treatment (see Table 4). Contrary to our third hypothesis, these findings suggest that the patients' and/or their families' choice to complete, or not to enter or complete post-hospitalization daycare program, was not based on the severity of their ED and comorbid psychiatric condition at admission to, or discharge from, inpatient treatment (it should be noted that all patients were offered to continue with daycare treatment if possible by the team of the inpatient department).

Second, most patients in both groups have normal BMI and regular menstrual periods at follow-up. The increase in BMI is achieved during inpatient treatment and maintained at 1-year follow-up, regardless of completing or not attending/not completing daycare treatment. These findings, shown also in our previous study of a different cohort of inpatients (25), are in keeping with follow-up studies showing that the discharge of inpatients with AN when reaching their required weight is associated with lower rate of relapse and rehospitalization in comparison with patients released before reaching their target weight range (54, 55).

In addition, social functioning has been rated as good or very good by a half of the participants, and bad or very bad by the other half of both groups. This somewhat unfavorable finding is of importance, as difficulties in social adjustment may persist in patients remitted from their ED (26), likely interfering with their remission and their overall adjustment (26, 56). Thus, in our previous study, disturbed social functioning at follow-up has been associated with a lower rate of remission, and with higher rates of post-discharge psychiatric comorbidity and rehospitalization (25).

Our results of adequate BMI regardless of not completing daycare program might raise a doubt about the necessity of this treatment in adolescent patients with EDs following long-term inpatient treatment. However, clinical interviews (such as the EDFHI) have been found to be more accurate in the prediction of remission from AN than the sole measure of BMI (25–27, 57–59). Thus, in our study, when looking at a more composite description of remission, a significant difference has been found between former inpatients with EDs treated or not treated in a post-hospitalization comprehensive daycare program for at least 5 months. The finding that almost half of the former inpatients treated in this facility are considered remitted from their ED at 1-year post-discharge using the strict Strober's remission criteria is striking. Other studies assessing the outcome of adolescents with AN following inpatient treatment (as are most of our patients; only 3/61 have been diagnosed with BN), but not providing post-hospitalization daycare, have found a remission rate at 1–2 years follow-up of around 10–30% (8, 9, 26, 60). The findings of the present research add to our previous study, which showed a tendency toward lower rehospitalization rate at 1-year follow-up in those patients treated with daycare. They also add to a previous naturalistic study in adolescents with AN treated in an ambulatory setting, showing that patients terminating treatment prematurely show decreased likelihood of achieving remission (57).

It is of further note that the other variable distinguishing between patients completing and not entering/not completing daycare treatment at 1-year follow-up was the greater percentage of continuation of psychotherapy among the completers (see Table 3). This finding may suggest a greater overall motivation to recover and to receive treatment among the completers. It is consistent with the notion that the motivation to recover and the cooperation of adolescents with EDs with their daycare treatment is essential for their remission (13, 15, 20). Thus, Green et al. (61) have shown that high initial motivation to change in daycare-treated adolescents with AN is associated with greater increase in BMI. Moreover, De Jong et al. (62) suggest that ED patients with better recovery outcomes are less likely to drop out of psychotherapeutic treatment vs. more severely ill patients. This finding is of importance, as the inclination of adolescents with EDs is usually to be less cooperative with their treatment (19, 24). The individual therapy of most patients of both groups after discharge is psychodynamic psychotherapy, perhaps because it has been the main individual psychotherapeutic mode during inpatient treatment. It adds to the findings of other daycare programs in patients with AN using psychodynamic psychotherapy as their main treatment, with favorable results (38). Most of the patients not attending daycare also continued with nutritional counseling.

In addition, the continuation of psychotherapy might have been particularly required for those remitted patients included in Strober's category (2), i.e., that although being remitted behaviorally, have still demonstrated maladaptive eating-related preoccupations at follow-up (12 of the 22 remitted patients have been included in this category). This finding is crucial, as the presence of ED-related and body image-related concerns following remission may be associated with a greater risk of relapse (28, 63, 64). It is certainly of relevance in our patients that, although released from inpatient treatment with a normal range BMI, and despite the symptomatic improvement achieved from admission to discharge (except for BIS), have still demonstrated eating-elated pathology (on the EAT-26) and disturbed attitudes and behaviors toward their body (BSI), as well as comorbid symptoms of depression (BDI) and anxiety (STAI; see Table 4). These comorbid symptoms have persisted, although most patients have been treated at discharge with SSTIs.

The overall symptomatic changes found from admission to discharge support the notion that improvement in ED-related symptoms may occur alongside a similar improvement in comorbid depressive and anxiety symptoms (26, 60, 65). Nonetheless, the continuation of both depressive and anxiety symptoms may interfere with the patients' later overall adjustment and increase the risk of ED-related relapse (25, 26, 60, 64, 65) and rehospitalization (66).

Whereas, a difference has been found between adolescents staying in the daycare program and those who have not in the consistency of psychotherapeutic treatment, no between-group difference has been found in the continuation of psychopharmacotherapy, with both groups showing high adherence. At the start, the fact that most adolescents have had evidence of comorbid depression and anxiety symptoms at discharge could have led the patients in both groups to continue with psychopharmacotherapy. Second, this finding is consistent with studies showing that most ED patients seeking treatment request psychopharmacological treatment, rather than psychotherapy (67). Nonetheless, in contrast to our findings, Halmi et al. (58) have found that patients with AN dropping out from treatment tend to abandon pharmacological treatment rather than psychotherapy.

### Limitations and Advantages

The findings of the present study should be interpreted with caution and regarded as preliminary because of several limitations. At the start, the size of the sample was relatively small, not enabling us to evaluate whether different types of EDs (AN, BN, or EDNOS) would differentially benefit from post-hospitalization daycare treatment. Second, the noncompleting group represented a heterogenous population, consisting of patients not entering treatment, and other patients not completing 5 months in daycare. Third, as noted earlier, only about a half of the girls filled the self-rating questionnaires at follow-up, not enabling the inclusion of the follow-up data in the ANOVA with repeated measures analysis. Fourth, the study was naturalistic rather than controlled with respect to the patients continuing or not entering/not continuing with post-hospitalization daycare. Nonetheless, as such, it likely resembled real-life conditions of treatment. Fifth, in contrast to other studies (3, 8, 9), inpatient treatment was relatively long, likely influencing the condition of the patients also at the daycare facility. Moreover, the daycare program itself was relatively long in comparison with other studies (3, 13, 61, 68–70). Nevertheless, as such, it provided an opportunity to assess the merit of long-term structured highly supervised inpatient and daycare programs in increasing the overall favorable outcome of the ED in relatively severely ill adolescents. Furthermore, short-term daycare programs were found to be associated with only modest weight gain (13, 61, 70) in comparison with the increase and maintenance of a mean BMI of around 3.4 kg/m^2^ from admission to 1-year follow-up. Sixth, the relatively long hospitalization period might have interfered with the social functioning of about half of our patients at follow-up, although both inpatient and daycare treatment were specifically geared toward its improvement. In addition, the follow-up period in our study was relatively short. Therefore, we plan to continue with a longer follow-up of our sample. Last, social functioning was assessed with open-ended questions rather than with a standardized tool, and we did not evaluate occupational functioning, as most of our patients in both groups returned to their school following discharge. This paradigm was also used in a previous study of our group (25).

Our study has, nevertheless, some important advantages. First, it adds to the limited literature about the clinical relevance of post-hospitalization halfway out daycare treatment for adolescent ED. Second, we have used a prospective longitudinal design, employing adequate clinical measures and structured follow-up assessment. Similar to some other studies (26, 27, 59), we have used comprehensive interview-based assessments of remission from an ED. Third, in contrast to many studies using self-report of weight, our patients have been weighed at the follow-up assessment. Last, all follow-up interviews have been conducted face to face.

### Recommendations for Future Research

First, future research should be conducted in larger populations and for longer periods, to find out whether the favorable 1-year post-hospitalization outcome of adolescents with EDs treated in a model of long inpatient and daycare treatment would be replicated in a larger sample and be maintained also at longer follow-up. Nevertheless, as the long-term outcome of an ED in adolescents is usually more favorable than the short outcome (26, 57, 59), we can expect the continuation of the better outcome of our daycare patients also in the long-run. Second, this research should be controlled, rather than naturalistic, as has been the case in some other studies of post-hospitalization daycare program (3). Third, our findings suggest that despite the recommendations not to release adolescent inpatients with EDs before reaching and maintaining their target weight, and before becoming asymptomatic regarding their ED behaviors (54, 55), the use of an adequate post-hospitalization daycare program might enable an earlier discharge from inpatient treatment. This is line with Herpertz-Dahlmann et al. (3) and Hay et al. (71) suggesting that daycare program after short inpatient care in adolescent patients with non-chronic AN seems no less effective than inpatient treatment for weight restoration and maintenance during the first year after admission. Fourth, our findings suggest that post-hospitalization daycare programs should be focused also on the management of comorbid psychiatric disorders and overall psychosocial functioning. Last, previous studies have emphasized the impact of familial cooperation in daycare programs on treatment outcome (7, 68, 72). Thus, in a setting like an adolescent daycare program, which is likely less structured than inpatient treatment, cooperation with parents becomes even more critical and should be assessed in future studies. In this respect, parents in our setting have often stated that the most meaningful work for them has begun following the discharge from inpatient treatment, when their daughters have returned home.

## Conclusion

The aim of the present study was to examine the contribution of a halfway out daycare program to the treatment of adolescents hospitalized because of an ED. Our findings indicated that a good post-discharge 1-year outcome from the ED was achieved when using a single criterion such as weight, even in former patients not continuing with daycare treatment. By contrast, when using more comprehensive criteria for the definition of remission such as the Strober's criteria (26), relating, at least in part, also to ED-related preoccupations and attitudes, less than a quarter of former inpatients not entering/not completing daycare program achieved remission in comparison with almost a half of the completers. This difference might be attributed, in part, to a greater inclination of completers to continue with psychotherapy following discharge.

## Data Availability Statement

The raw data supporting the conclusions of this article will be made available by the authors, without undue reservation.

## Ethics Statement

The studies involving human participants were reviewed and approved by Helsinki Committee, Sheba Medical Center, Tel Hashomer, Israel. Written informed consent to participate in this study was provided by the participants' legal guardian/next of kin.

## Author Contributions

LL-C, AY, and DS contributed to the conception, design of the study, and were responsible for the organization of the article. All authors contributed to the follow-up of the patients, the analysis of the data, read and approved the final draft of this article.

## Conflict of Interest

The authors declare that the research was conducted in the absence of any commercial or financial relationships that could be construed as a potential conflict of interest.
